# The hypoperfusion volume has a strong predictive value for hemorrhagic transformation in acute ischemic stroke patients with anterior circulation occlusion after endovascular thrombectomy

**DOI:** 10.1186/s12883-025-04186-5

**Published:** 2025-04-16

**Authors:** Danxia Chen, Bingdong Xu, Tongguo Wei, Qinhui Zhu, Yu Zhong, Yusheng Zhang

**Affiliations:** 1 Department of Neurology, Meizhou People’s Hospital, Meizhou, Guangdong China; 2 Department of Neurology, The First Affiliated Hospital of Jinan University, Guangzhou, Guangdong P.R. China

**Keywords:** Endovascular thrombectomy, Hypoperfusion volume, Symptomatic intracerebral hemorrhage, Intracerebral hemorrhage, Acute ischemic stroke

## Abstract

**Objective:**

It remains unclear whether hypoperfusion volume elevates the risk of hemorrhagic transformation (HT) after endovascular thrombectomy (EVT) in patients with acute ischemic stroke (AIS). This study aims to investigate the association between hypoperfusion volume and HT after EVT.

**Materials and methods:**

We retrospectively recruited AIS patients with anterior circulation occlusion after receiving EVT from January 2021 to May 2024. The pre-EVT hypoperfusion volume was assessed using computed tomography perfusion, with a hypoperfusion area defined as time-to-maximum > 6s. Multivariable analysis determined whether the hypoperfusion volume served as an independent predictor of symptomatic intracerebral hemorrhage (sICH) or intracerebral hemorrhage (ICH), and its predictive value was evaluated using receiver operating characteristic (ROC) curves.

**Results:**

A total of 115 patients were analyzed, with 55 (47.8%) having ICH and 16 (13.9%) experiencing sICH. The median age was 67, and 28.6% were female. The median core infarct volume was 4.3 ml, and the median hypoperfusion volume was 112.8 ml. After adjusting for potential confounding factors, the hypoperfusion volume remained independently correlated with sICH (OR = 1.008, 95% CI = 1.001–1.015, *P* = 0.018) and ICH (OR = 1.006, 95% CI = 1.001–1.012, *P* = 0.033). ROC curve analysis demonstrated that the hypoperfusion volume effectively predicted sICH [(area under the curve (AUC) = 0.702] or ICH (AUC = 0.643).

**Conclusion:**

The hypoperfusion volume has a strong predictive value for sICH and ICH in AIS patients with anterior circulation occlusion after EVT. This underscores the necessity of assessing the hypoperfusion volume before EVT, particularly for patients with smaller core infarct volumes in AIS.

## Introduction

In 2019, the global incidence of stroke reached 12.2 million cases, impacting over 101 million individuals. Stroke contributed to a total of 143 million disability-adjusted life years, resulting in 6.55 million deaths attributed to stroke [1]. This positions stroke as the second leading cause of death worldwide and the third major contributor to the combined burden of death and disability [2]. Globally, clinical guidelines widely endorse endovascular thrombectomy (EVT) for its efficacy in enhancing long-term clinical outcomes in acute ischemic stroke (AIS) patients [3–5]. Nevertheless, it is an indisputable reality that the success rate of EVT remains approximately 50% [6], with a higher risk of postoperative hemorrhagic transformation (HT) [7], notably parenchymal hemorrhage (PH) [8, 9] and symptomatic intracerebral hemorrhage (sICH) [10], contributing to a poorer prognosis.

Cerebral reperfusion injury represents a significant complication arising from mechanical thrombectomy in the treatment of AIS [11]. It results in energy failure and cell death within affected tissues by impacting adenosine triphosphate production in the mitochondria of brain cells [12]. HT stands out as a prominent radiographic manifestation of cerebral reperfusion injury [13]. Typically, a larger area of hypoperfusion indicates an elevated risk of cerebral reperfusion injury and the subsequent onset of intracerebral hemorrhage (ICH) upon the restoration of cerebral blood flow (CBF). Fundamental research has unveiled that prolonged administration of recombinant tissue plasminogen activator (rt-PA) in mice with unilateral middle cerebral artery occlusion leads to increased blood-brain barrier (BBB) permeability to hemoglobin, consequently heightening the risk of HT [14]. Substantiated by clinical studies, BBB permeability emerges as a predictive factor for HT following acute reperfusion therapy [15]. Consequently, accurately predicting HT becomes of paramount significance through the assessment of the degree of cerebral reperfusion injury.

Early prediction of HT is crucial for neurologists to implement measures to prevent clinical deterioration and formulate optimal treatment plans. Presently, post-processing software for automatic perfusion commonly employs time-to-maximum (Tmax) > 6 s as the threshold for identifying ischemic hypoperfusion areas [16, 17]. The magnitude of the hypoperfusion volumes, to some extent, mirrors the severity of cerebral ischemia-reperfusion injury following EVT. This study utilized computed tomography perfusion (CTP) to compute the cerebral hypoperfusion volumes in patients with AIS and investigated whether the hypoperfusion volumes can autonomously predict HT, along with assessing the accuracy of this prediction.

## Methods

### Study design and participants

We conducted a retrospective recruitment of patients with AIS admitted to Meizhou People’s Hospital, Guangdong, China, between January 2021 and May 2024. All data were extracted from electronic health record systems and imaging data systems, which were recorded and analyzed by trained healthcare professionals. Inclusion criteria were as follows: age over 18 years; admission to the hospital within 24 h of symptom onset; pre-onset modified Rankin Scale score of 1 or lower; confirmation of anterior circulation occlusion including internal carotid artery and/or middle cerebral artery, by computed tomography angiography with CTP assessment of hypoperfusion volume; undergoing EVT, with postoperative modified thrombolysis in cerebral infarction (mTICI) 2b/3. Exclusion criteria were as follows: preoperative CT confirmation of cerebral hemorrhage or subarachnoid hemorrhage; insufficient image quality for data analysis; missing baseline data. The study was conducted in accordance with the ethical standards outlined in the 1975 Declaration of Helsinki and was approved by the institutional review board of Meizhou People’s Hospital (approval number: 2023-C-94, date of approval: 28 September 2023). Written informed consents of patients were waived according to a protocol approved by the institutional review committee. Additionally, we adhered to the guidelines outlined in the Strengthening the Reporting of Observational studies in Epidemiology statement to ensure comprehensive and transparent reporting of the study.

### Clinical evaluation

Baseline demographic data, including age, gender, risk factors such as diabetes, hypertension, hyperlipidemia, and atrial fibrillation, intravenous thrombolysis, time from onset to recanalization, preprocedural National Institutes of Health Stroke Scale (NIHSS), preprocedural Alberta Stroke Program Early Computed Tomography Score (ASPECTS), and blood biochemical data, were all recorded in our database. Postoperative mTICI scores were documented and evaluated by neurologist W.T.G with over 10 years of experience.

A repeat brain CT scan was conducted 22–36 h after EVT or earlier in cases of early neurological deterioration after EVT. sICH was defined as any hemorrhage accompanied by a neurological deterioration of 4 or more points on the NIHSS from baseline [18]. HT grading was based on the European Cooperative Acute Stroke Study (ECASS) II classification, which included hemorrhagic infarction (HI)-1, HI-2, parenchymal hemorrhage (PH)-1, and PH-2 [19].

### Measurement of infarct core and hypoperfusion volume

All patients underwent dual-source CT scans (Siemens, SOMATOM Force) for examination. During the scan, patients were placed in a supine position with their heads first, and a non-ionic iodine contrast agent (Iopamidol 370 mgI/mL) was injected through the elbow vein. The CT plain scan included a whole cranial volume scan, while the cranial CTP scan used automatic tracking triggering technology. In the cranial CTP scan, 30 ml of contrast agent was injected through the elbow vein at a speed of 5 ml/s, followed by an injection of 30 ml of 0.9% sodium chloride injection solution. The cranial CTP scan had a tube voltage of 80 kV, tube current of 200mAs, slice thickness of 1.2 mm, and a scan time of 45.45 s. After the cranial CTP scan, a head and neck CT angiography (CTA) examination was performed approximately 5 min later. The head and neck CTA scan range extended from the aortic arch to the top of the skull and used automatic tracking triggering technology. In the head and neck CTA scan, 40 ml of contrast agent was injected through the elbow vein at a speed of 4.5 ml/s, followed by an injection of 30 ml of 0.9% sodium chloride injection solution. The head and neck CTA scan had a tube voltage of 100 kV, tube current of 160mAs, slice thickness of 0.6 mm, and a scan time of 2.5 s.

In this study, CBF less than 30% was defined as the core infarct area, and a Tmax > 6s was defined as the hypoperfusion area [20]. The core infarct volume and hypoperfusion volume were automatically calculated using the combined United Imaging Artificial Intelligence software.

### Statistical analysis

Statistical analysis was conducted using SPSS 26.0 software (SPSS Inc., Chicago, IL, USA). Normality of data distribution was assessed using the Kolmogorov-Smirnov test. Data conforming to a normal distribution were expressed as mean ± standard deviation, while non-normally distributed data were presented as median and interquartile range (IQR). Count data were expressed as frequency and percentage. Spearman correlation analysis was performed to explore the association between hypoperfusion volume and sICH or ICH. Univariable and multivariable logistic regression analyses were employed to investigate whether the hypoperfusion volume was an independent predictor of sICH or ICH. The multivariable logistic regression model used a forward stepwise method to select variables in descending order of their association strength with the outcomes (*P* ≤ 0.05 to enter, *P* > 0.1 to exit). The diagnostic performance of the logistic regression model was further evaluated by calculating accuracy, sensitivity, specificity, positive predictive value (PPV), and negative predictive value (NPV) based on the optimal cutoff value determined by the receiver operating characteristic (ROC) curve analysis. To evaluate the goodness of fit of the model, the Hosmer-Lemeshow test was employed. Additionally, tolerance and variance inflation factor (VIF) were calculated for each covariate to assess collinearity within the final multivariable model. ROC curve analysis was performed to assess the predictive value of the hypoperfusion volume for sICH or ICH, estimating the area under the curve (AUC) and its 95% confidence interval. A *P* value < 0.05 was considered statistically significant.

## Results

### Baseline results

**Fig. 1:**
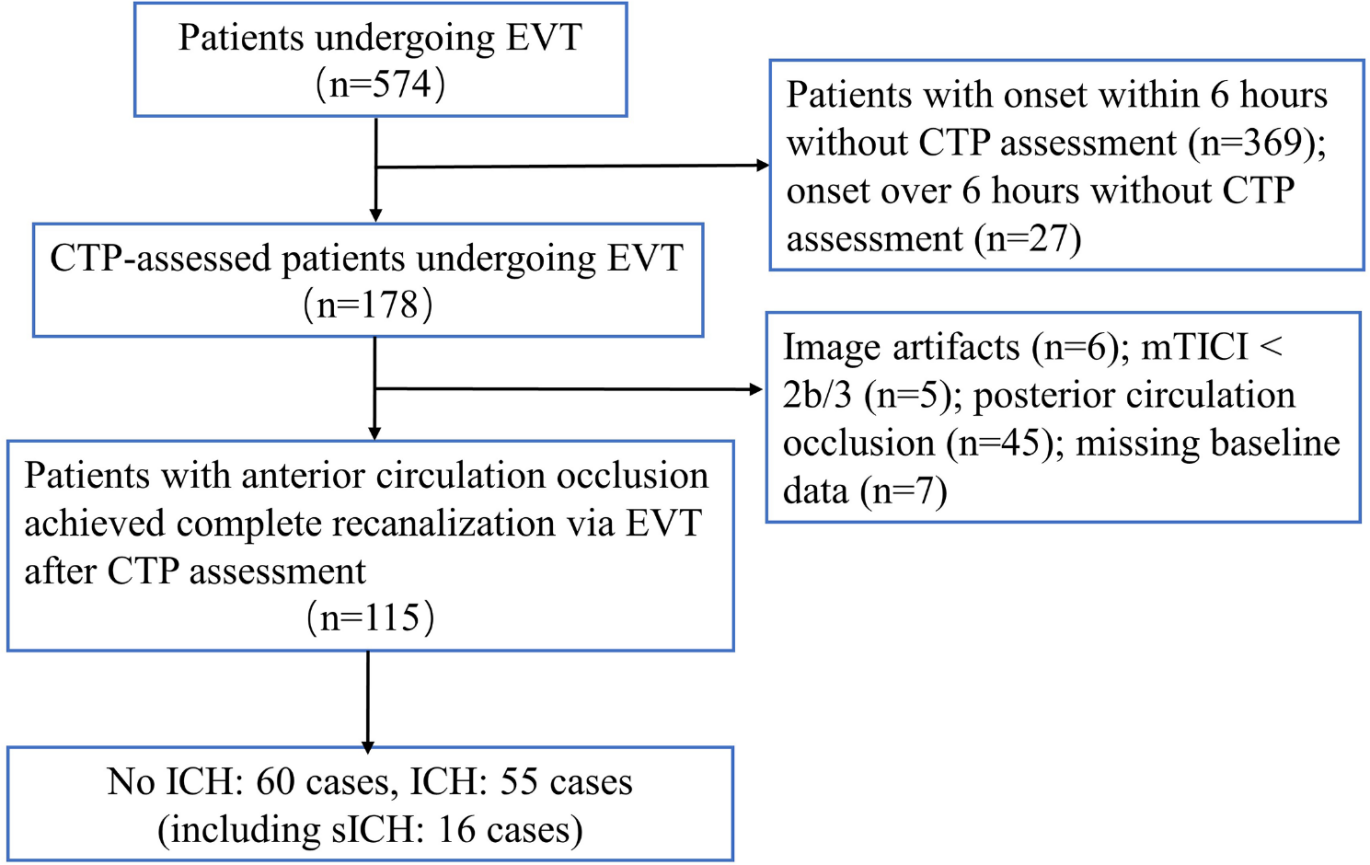
The flow chart of the study. EVT, Endovascular Thrombectomy; CTP, Computed Tomography Perfusion; mTICI, modified Thrombolysis in Cerebral Infarction; sICH, Symptomatic Intracerebral Hemorrhage; ICH, Intracerebral Hemorrhage

A total of 574 patients underwent EVT treatment, among whom 178 were assessed for hypoperfusion volumes using CTP. After excluding patients with image artifacts, mTICI < 2b/3, posterior circulation occlusion, and baseline data missing, a final cohort of 115 patients was included for subsequent analysis (see Fig. 1). The included cohort exhibited a median age of 67 years (IQR, 57.0–74.0 years), with females constituting 28.6%. On admission, the median NIHSS score was 12 (IQR, 8.0–16.0), and the median ASPECTS score was 8 (IQR, 7.0–9.0). Systolic and diastolic blood pressures had median values of 151.5 mmHg and 88 mmHg, respectively. Among the participants, 69.5% had a history of hypertension, 26.9% had a history of diabetes mellitus, and 20.0% were diagnosed with atrial fibrillation. Additionally, the median time from onset to door was 717.5 min, the median time from onset to recanalization was 786.0 min, and the median number of thrombectomy procedures was one. The median core infarct volume was 4.3 ml (IQR, 0.6–14.3 ml), and the median hypoperfusion volume was 112.8 ml (IQR, 75.9–164.4 ml). sICH occurred in 13.9% of cases, and ICH occurred in 47.8%. Additional baseline characteristics of all participants were presented in Table 1.

**Table 1:** Baseline characteristics

Demographic variables	Patients (*n* = 115)
Age (y), median (IQR)	67.0 (57.0–74.0)
Female, n (%)	33 (28.6)
Baseline NIHSS, median (IQR)	12.0 (8.0–16.0)
Baseline ASPECTS, median (IQR)	8.0 (7.0–9.0)
Medical history	
Hypertension, n (%)	80 (69.5)
Diabetes, n (%)	31 (26.9)
Hyperlipidemia, n (%)	27 (23.4%)
Atrial fibrillation, n (%)	23 (20.0)
Smoking, n (%)	18 (15.6)
Obesity, n (%)	25 (21.7)
Anticoagulant therapy before AIS, n (%)	6 (5.2)
Intravenous alteplase treatment, n (%)	13 (11.3)
Systolic blood pressure (mmHg), median (IQR)	151.5 (140.0–164.0)
Diastolic blood pressure (mmHg), median (IQR)	88.0 (78.7–98.0)
Vascular occlusion site, n (%)	
ICA	11 (9.6)
MCA	83 (72.1)
ICA + MCA	21 (18.3)
Stroke etiology, n (%)	
Atherosclerosis	74 (64.3)
Cardioembolic	40 (34.8)
Other	1 (0.9)
Onset to door (min), median (IQR)	717.5 (437.2–973.0)
Onset to recanalization (min), median (IQR)	786.0 (492.0–1113.7)
Number of passes, median (IQR)	1.0 (1.0–2.0)
Laboratory testing	
Platelet count, median (IQR)	210.0 (164.0–242.0)
INR, median (IQR)	0.9 (0.9–1.1)
APTT, median (IQR)	33.5 (31.3–36.5)
D-dimer, median (IQR)	0.5 (0.3–1.4)
ALT, median (IQR)	17.0 (13.0–25.0)
AST, median (IQR)	20.0 (17.0–24.7)
HbA1C (%), median (IQR)	6.1 (5.7–6.8)
Infarct core volume (ml), median (IQR)	4.3 (0.6–14.3)
Hypoperfusion volume, median (IQR)	112.8 (75.9–164.4)
sICH	16 (13.9)
ICH	
No	60 (52.2)
HI-1	16 (13.9)
HI-2	17 (14.8)
PH-1	15 (13.0)
PH-2	7 (6.1)

Abbreviations: NIHSS, National Institutes of Health stroke scale; ASPECTS, Alberta Stroke Program Early CT Score; AIS, Acute Ischemic Stroke; ICA, Internal Carotid Artery; MCA, Middle Cerebral Artery; HI, Hemorrhagic Infarction; PH, Parenchymal Hemorrhage; sICH, Symptomatic Intracerebral Hemorrhage; INR, International Standard Ratio; APTT, Activated Partial Thromboplastin Time; ALT, Alanine Transaminase; AST, Aspartate Transaminase; HbA1C, Hemoglobin A1c; IQR, Median and Interquartile Range

**Fig. 2:**
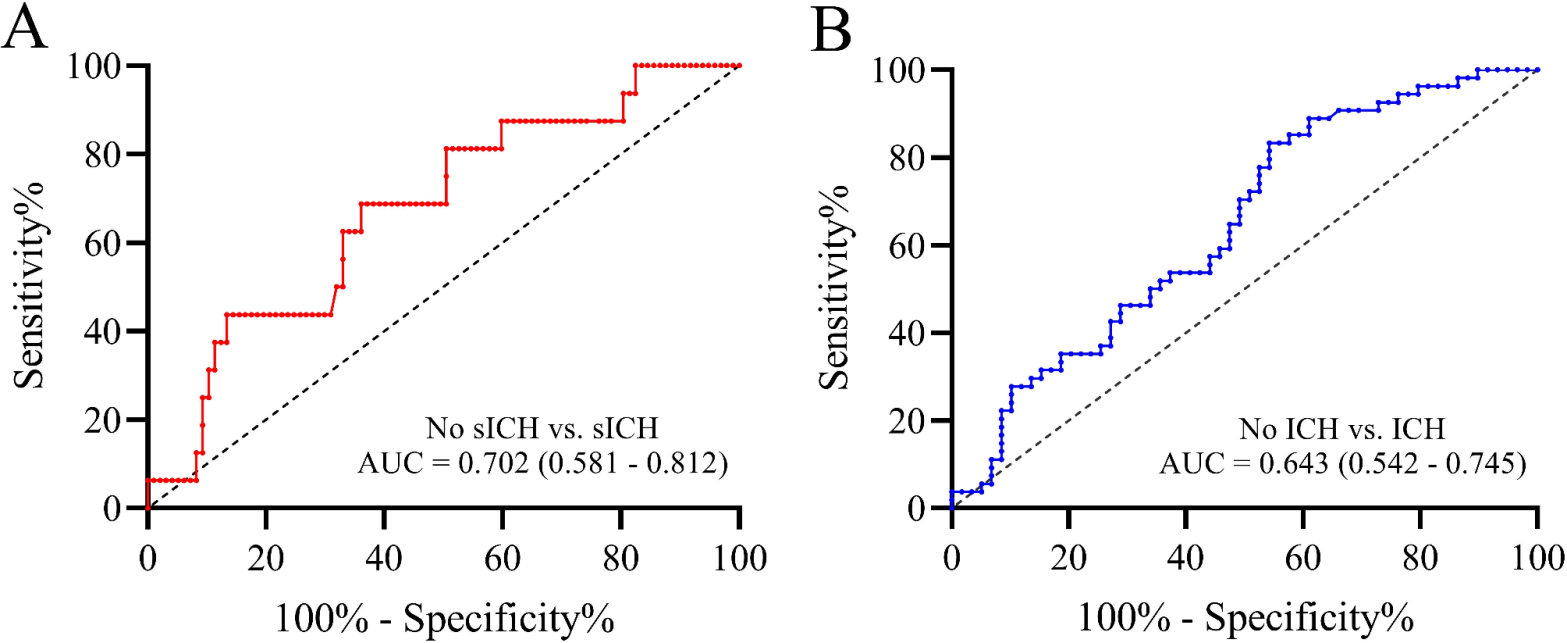
Receiver operating characteristic curves with the hypoperfusion volume independently associated with sICH and ICH. sICH, Symptomatic Intracerebral Hemorrhage; ICH, Intracerebral Hemorrhage

During the in-hospital period, no mortality events were observed. However, at the 90-day follow-up, three patients had died, including one due to a neurological cause (recurrent severe cerebral infarction leading to brain herniation) and two due to non-neurological causes (severe heart failure and disseminated intravascular coagulation, respectively).

### Predictive values of the volume for sICH and ICH

The outcomes of univariable analysis unveiled that the individuals with larger hypoperfusion volumes faced an increased risk of sICH (OR = 1.007, 95% CI = 1.002–1.014, *P* = 0.027). After adjusting for baseline systolic blood pressure, a noteworthy association persisted (OR = 1.008, 95% CI = 1.001–1.015, *P* = 0.018) (see Table 2). The model demonstrated an accuracy of 0.714, with a sensitivity of 68.7%, specificity of 72.6%, PPV of 43.9%, and NPV of 92.4%. The results of the Hosmer-Lemeshow test were not significant (*P* = 0.584), indicating a good model fit. The tolerance for covariates in the final multivariable model ranged from 0.96 to 0.99, and the VIF ranged from 1.11 to 1.14. Similarly, univariable analysis demonstrated that a larger hypoperfusion volume correlated with an elevated risk of ICH (OR = 1.007, 95% CI = 1.001–1.012, *P* = 0.019). Following adjustment for other significant predictors of ICH, hypoperfusion volume remained an independent predictor (OR = 1.006, 95% CI = 1.001–1.012, *P* = 0.033) (see Table 3). This model achieved an accuracy of 0.692, with a sensitivity of 72.2%, specificity of 61.0%, PPV of 62.9%, and NPV of 70.6%. The Hosmer-Lemeshow test yielded a non-significant result (*P* = 0.236), suggesting a well-fitted model. The covariates in the final multivariable model exhibited tolerances ranging from 0.62 to 0.90, with VIF values ranging from 1.18 to 1.94.

**Table 2:** Univariable and multivariable logistic regression analyses of risk factors for sICH

Parameter	Univariable analysis OR (95% CI)	*P* value	Multivariable analysis OR (95% CI)	*P* value
Age	1.020 (0.973–1.070)	0.403		
Female	0.877 (0.260–2.955)	0.832		
Baseline NIHSS	1.071 (0.986–1.164)	0.105		
Baseline ASPECTS	0.716 (0.465–1.100)	0.127		
**Medical history**				
Hypertension	0.700 (0.233–2.107)	0.526		
Diabetes	0.877 (0.260–2.955)	0.832		
Hyperlipidemia	0.185 (0.023–1.468)	0.110		
Atrial fibrillation	1.368 (0.402–4.777)	0.605		
Smoking	1.246 (0.316–4.909)	0.753		
Obesity	1.222 (0.357–4.182)	0.749		
Intravenous alteplase treatment	0.709 (0.142–3.545)	0.675		
Systolic blood pressure	1.029 (1.001–1.057)	0.039^*^		
Diastolic blood pressure	1.022 (0.978–1.067)	0.341		
Onset to door	1.000 (1.000–1.000)	0.402		
Onset to recanalization	1.000 (1.000–1.000)	0.335		
Number of passes	1.328 (0.795–2.219)	0.278		
**Laboratory testing**				
Platelet count	0.995 (0.986–1.004)	0.271		
INR	0.976 (0.789–1.206)	0.819		
APTT	1.033 (0.966–1.105)	0.342		
D-dimer	1.020 (0.841–1.236)	0.841		
ALT	1.002 (0.956–1.051)	0.935		
AST	1.002 (0.967–1.038)	0.915		
HbA1C	0.624 (0.322–1.208)	0.162		
Infarct core volume	1.011 (0.994–1.028)	0.197		
Hypoperfusion volume	1.007 (1.002–1.014)	0.027^*^	1.008 (1.001–1.015)	0.018^*^

Abbreviations: Abbreviations: NIHSS, National Institutes of Health stroke scale; ASPECTS, Alberta Stroke Program Early CT Score; sICH, Symptomatic Intracerebral Hemorrhage; INR, International Standard Ratio; APTT, Activated Partial Thromboplastin Time; ALT, Alanine Transaminase; AST, Aspartate Transaminase; HbA1C, Hemoglobin A1c; IQR, Median and Interquartile Range; OR, Odds Ratio; CI, Confidence Interval

*P < 0.05

**Table 3:** Univariable and multivariable logistic regression analyses of risk factors for ICH

Parameter	Univariable analysis OR (95% CI)	*P* value	Multivariable analysis OR (95% CI)	*P* value
Age	0.990 (0.958–1.022)	0.533		
Female	0.488 (0.208–1.144)	0.099		
Baseline NIHSS	1.091 (1.014–1.173)	0.019^*^	1.081 (1.003–1.165)	0.041^*^
Baseline ASPECTS	0.692 (0.492–0.971)	0.033^*^		
**Medical history**				
Hypertension	0.504 (0.224–1.132)	0.097		
Diabetes	0.488 (0.208–1.144)	0.099		
Hyperlipidemia	0.358 (0.142–0.906)	0.030^*^		
Atrial fibrillation	1.517 (0.603–3.812)	0.376		
Smoking	1.420 (0.515–3.916)	0.498		
Obesity	0.804 (0.329–1.961)	0.631		
Intravenous alteplase treatment	1.221 (0.426–3.499)	0.711		
Systolic blood pressure	0.994 (0.976–1.013)	0.553		
Diastolic blood pressure	0.994 (0.964–1.024)	0.688		
Onset to door	1.000 (1.000–1.000)	0.695		
Onset to recanalization	1.000 (1.000–1.000)	0.686		
Number of passes	1.306 (0.868–1.964)	0.200		
**Laboratory testing**				
Platelet count	1.003 (0.997–1.009)	0.331		
INR	0.385 (0.014–10.806)	0.575		
APTT	0.969 (0.910–1.031)	0.317		
D-dimer	0.939 (0.795–1.110)	0.463		
ALT	1.004 (0.970–1.039)	0.827		
AST	1.014 (0.984–1.045)	0.363		
HbA1C	0.755 (0.578–1.068)	0.128		
Infarct core volume	1.016 (0.997–1.036)	0.101		
Hypoperfusion volume	1.007 (1.001–1.012)	0.019 ^*^	1.006 (1.001–1.012)	0.033^*^

Abbreviations: NIHSS, National Institutes of Health stroke scale; ASPECTS, Alberta Stroke Program Early CT Score; ICH, Intracerebral Hemorrhage; INR, International Standard Ratio; APTT, Activated Partial Thromboplastin Time; ALT, Alanine Transaminase; AST, Aspartate Transaminase; HbA1C, Hemoglobin A1c; IQR, Median and Interquartile Range; OR, Odds Ratio; CI, Confidence Interval

*P < 0.05

Moreover, Spearman correlation analysis revealed positive associations between hypoperfusion volume and both sICH (r_s_ = 0.211, *P* = 0.025) and ICH (r_s_ = 0.247, *P* = 0.009). Additionally, hypoperfusion volume exhibited a positive correlation with HT grades (r_s_ = 0.279, *P* = 0.003).

Furthermore, hypoperfusion volume exhibited a significant diagnostic capability for postoperative sICH, with an AUC of 0.702 (95% CI, 0.592–0.812), and its optimal cutoff value was determined to be 128.8 ml. In this context, the sensitivity and specificity for diagnosing sICH were 68.7% and 72.6% respectively (see Fig. 1A). Similarly, hypoperfusion volume demonstrated diagnostic capability for postoperative ICH, presenting an AUC of 0.643 (95% CI, 0.542–0.745), and its optimal cutoff value was determined to be 85.0 ml. The sensitivity and specificity for diagnosing ICH were 83.3% and 46.5% respectively (see Fig. 1B).

## Discussion

In this retrospective single-center study, we validated the independent impact of hypoperfusion volume on sICH and ICH in patients with AIS undergoing EVT. We also demonstrated its robust predictive capability for postoperative sICH and ICH. Additionally, we observed a modest positive correlation between low perfusion volume and sICH, ICH, and HT grade. Therefore, routine assessment of hypoperfusion volume may be necessary. This aids clinicians in considering appropriate adjustments to treatment plans, including closer postoperative monitoring and cautious anticoagulant therapy.

The success of the DEFUSE-3 study has highlighted the importance of preoperative assessment of cerebral hypoperfusion volume [20]. However, it is crucial to note that the larger the hypoperfusion area, the greater the severity of post-ischemic reperfusion injury and the associated risk of HT [21]. Our study results showed that even after adjusting for admission systolic blood pressure, hypoperfusion volume remained an independent risk factor for sICH (OR = 1.008) and ICH (OR = 1.006), and hypoperfusion volume exhibited a positive correlation with HT grade. This means that for every 10-milliliter increase in hypoperfusion volume, the risk of sICH and ICH increased by 8% and 6% respectively. This emphasizes the clinical utility of hypoperfusion volume as an independent risk factor, providing clinicians with a more precise predictive tool to identify the risk of sICH and ICH occurrence among stroke patients. Through the assessment of hyper-perfusion using arterial spin labeling, a robust independent correlation between high CBF and subsequent HT was observed [22], and the longer the duration of hyper-perfusion, the higher the HT grade [23], aligning with our study results. In contrast to earlier studies, we utilized hypoperfusion volume calculated based on CTP to evaluate the perfusion status. In comparison to arterial spin labeling, CTP offers imaging stability in assessing cerebral perfusion and is less prone to overestimating cerebral hypoperfusion, rendering it more favorable in related clinical trials [20, 24]. Furthermore, earlier studies primarily employed CTP parameters such as CBF and cerebral blood volume [25], to assess cerebral perfusion status, whereas our study utilized equivalent parameters to ascertain whether Tmax > 6s surpassed the ischemic perfusion threshold, offering a more intuitive assessment of cerebral perfusion status.

This study also made an intriguing discovery concerning the predictive capability of hypoperfusion volume for postoperative sICH and ICH with high accuracy. The AUC for sICH was 0.702 (optimal cutoff value: 128.8 ml), exceeding the AUC of 0.677 (optimal cutoff value: 85.0 ml) for predicting ICH. Additionally, hypoperfusion volume exhibited a high specificity of 72.6% for predicting sICH but a lower sensitivity. Conversely, hypoperfusion volume demonstrated a high sensitivity of 83.3% for predicting ICH but a lower specificity. This implies that for patients with AIS necessitating EVT treatment, employing a cutoff value of 85.0 ml for hypoperfusion volume as the diagnostic criterion to identify the occurrence of postoperative ICH results in a low rate of missed diagnoses. Similarly, employing a cutoff value of 128.8 ml for hypoperfusion volume as the diagnostic criterion to identify the occurrence of postoperative sICH results in a low rate of misdiagnoses. This holds significant implications for clinicians in formulating post-acute AIS antiplatelet or anticoagulation treatment plans.

Multiple physiological mechanisms can induce ischemia, resulting in hypoxia and inadequate blood flow, such as atherosclerosis, triggering anaerobic metabolism and impairing the mitochondrial electron transport chain [26]. However, restoring blood flow through treatment might inflict additional harm to ischemic tissue, a phenomenon known as ischemia-reperfusion injury [27]. During the reperfusion phase, mitochondrial damage and electrolyte imbalance can foster oxidative stress, culminating in cellular injury and death. Therefore, patients with AIS unavoidably undergo cerebral ischemia-reperfusion injury following intravenous thrombolysis or EVT treatment. In cases of severe reperfusion injury, it can result in substantial disruption of the BBB, permitting peripheral blood infiltration into brain tissues, ventricles, and the subarachnoid space [28]. Notably, intraventricular extension (IVE) of hematoma, a well-established predictor of poor prognosis in thalamic hemorrhage [29], mechanistically aligns with our findings that hypoperfusion-induced BBB disruption mediates ventricular blood infiltration. This underscores IVE as a critical prognostic marker across stroke subtypes, warranting prioritized assessment in future studies. This phenomenon might elucidate why hypoperfusion volume independently influences HT and predicts its occurrence.

Previous studies have demonstrated an association between a larger core infarct volume, higher NIHSS score, and lower ASPECTS score, and an elevated risk of HT [30, 31]. However, the univariable logistic regression analysis in this study did not identify a significant correlation between these three factors and sICH, contradicting previous research findings [32]. One potential explanation for this inconsistency is that the median volume of low perfusion in this study (112.8 ml) significantly surpassed the core infarct volume (4.3 ml), indicating that cerebral ischemia-reperfusion injury predominantly contributes to HT, as opposed to the brain tissue damage resulting from the core infarct. The relatively modest core infarct volume in this study underscores the necessity for heightened vigilance concerning the risk of sICH arising from post-EVT cerebral ischemia-reperfusion injury in patients with small-area infarction and extensive hypoperfusion volume.

Nevertheless, it is crucial to recognize the limitations of our study. The primary limitations of this study lie in its retrospective and single-center design, which may lead to selection bias, the influence of confounding variables, and a relatively small sample size. We mitigated these biases by including all eligible patients and employing multivariable analysis. Future studies should focus on several critical areas: integrating hypoperfusion volume with advanced biomarkers such as BBB permeability for HT risk prediction; investigating the role of IVE and hematoma dynamics in post-EVT outcomes; validating findings through multicenter studies to improve generalizability; and testing hypoperfusion mitigation strategies, including hemodynamic optimization, in interventional trials. These efforts will confirm and extend our findings, facilitating their translation into clinical practice.

## Conclusions

The study establishes hypoperfusion volume as an independent factor influencing sICH and ICH in patients undergoing EVT for AIS. Moreover, it exhibits excellent predictive accuracy for postoperative sICH and ICH. The results underscore an increased risk of HT post-surgery in patients with a small-area infarction and extensive hypoperfusion volume. Thus, clinicians should exercise caution when deciding on the timing and dosage of antiplatelet and anticoagulant treatments for this patient cohort.

## Data Availability

The datasets analyzed in this study can be obtained from the corresponding author upon reasonable request.
